# Non-adaptive territory selection by a bird with exceptionally long parental care

**DOI:** 10.7717/peerj.1852

**Published:** 2016-03-24

**Authors:** Radosław Włodarczyk, Piotr Minias

**Affiliations:** Department of Biodiversity Studies and Bioeducation, University of Łódź, Łódź, Poland

**Keywords:** Territory selection, Mute swan, Cygnus olor, Reproductive success, Body condition, Occupancy

## Abstract

High-quality territories are expected to provide greater fitness return for breeding individuals and, thus, are likely to have higher long-term occupation rate in comparison to low-quality territories. However, if environmental and ecological cues used for territory selection cannot reliably predict true territory quality, a mismatch between preferences and fitness may occur. We suggest that this kind of non-adaptive territory selection is more likely in species with long reproductive cycles, as a long time interval between territory establishment and young fledgling should reduce predictability of conditions during the critical stages of brood care. In this study, we investigated adaptiveness of territory selection in a migratory bird with exceptionally long parental care, the mute swan *Cygnus olor*, which requires over four months to complete the entire reproductive cycle from egg laying to young fledging. For this purpose, we collected information on the long-term (10–19 years) occupancy of 222 swan breeding territories and correlated it with reproductive performance (n = 1,345 breeding attempts) and body condition of breeding adults. We found that long-term occupancy positively correlated with the timing of breeding, suggesting that individuals settled earlier in the attractive, frequently occupied territories. By contrast, we found no relationship between territory occupancy and reproductive output (hatching and fledging success) or adult body condition. The results indicate that at the time of territory selection swans might not be able to reliably assess territory quality, likely due to: (1) exceptionally long period of parental care, which reduces temporal correlation between the conditions at the time of territory selection and conditions during chick rearing; and (2) unpredictability of human-related activities that had a major impact on reproductive output of swan pairs in our population.

## Introduction

Resources, such as food or nest sites, are usually unevenly distributed in space, which forces organisms to judge habitat quality while making settlement decisions (Chalfoun & Martin, 2007). This is especially important during the process of breeding territory choice, as the quality of breeding habitat to a large extent determines individual fitness. Since high-quality territories are likely to enhance breeding success (Morris, 2003), they should be occupied earlier in the breeding season by high-quality, dominant individuals (Currie, Thompson & Burke, 2000; Forstmeier, 2002). Also, high-quality territories are expected to have a higher probability of being occupied in any given season, so they should have a higher long-term occupation rate in comparison to low-quality, suboptimal territories (Janiszewski, Minias & Wojciechowski, 2013). However, these predictions may only be valid providing that organisms are able to appropriately judge habitat quality (Martin, 1998).

Settlement decisions can rarely be based on direct assessment of fitness return (Storch & Frynta, 1999). Thus, territory selection often depends on actual environmental and ecological characteristics that can impact fitness, including food resources, nest site availability, predation pressure, or landscape structure (Martin, 1987; Aebischer et al., 1996; Eggers et al., 2005; Thomson et al., 2006; Farrell et al., 2012; Janiszewski et al., 2014a). Reliable environmental cues should reflect territory quality not only during settlement but also later on, when breeding is advanced (Chalfoun & Martin, 2007). This seems to be especially important for species with long reproductive cycles, in which families stay within breeding sites for several months. However, appropriate assessment of temporal environmental cues can be difficult in migratory species, which usually have a narrow time window to collect information on territory quality following their arrival on breeding grounds (Siikamäki, 1998). Assessment of territory quality may be additionally hampered in anthropogenic landscape, where natural environmental cues at the time of settlement may be unreliable due to unpredictability of human activities (Kokko & Sutherland, 2001; Robertson & Hutto, 2006). In such conditions, individuals may not be able to make optimal settlement decisions.

The aim of our study was to investigate adaptiveness of territory selection in a migratory bird with exceptionally long parental care, the mute swan *Cygnus olor*. The mute swan is a large, long-lived species (average lifespan of 8.1 years; Rees et al., 1996), which requires four months to complete the entire reproductive cycle from egg laying to young fledging (Wieloch, Włodarczyk & Czapulak, 2004). Consequently, we hypothesize that environmental and ecological cues used for territory selection at the time of settlement may not allow birds to reliably predict territory quality at the later stages of the breeding cycle. Also, mute swans across the entire European range often breed in semi-natural landscape with moderate or strong anthropogenic pressure (Wieloch, Włodarczyk & Czapulak, 2004), which may further decrease reliability of cues used for territory choice. As a result, we expected that mute swans may not be able to reliably assess territory quality and select breeding territories adaptively to maximize their fitness return. To test this hypothesis we collected information on the occupation rate of more than 200 swan breeding territories in central Poland over the period of 10–19 years. As preferred territories are expected to have higher occupation rate in comparison to less preferred territories (Sergio & Newton, 2003), long-term territory occupancy is considered a good indicator of territory attractiveness, i.e. of how individuals respond to habitat cues during territory selection. To test whether territory choice in the mute swan is adaptive, we correlated long-term territory occupancy with different reproductive components (timing of egg laying, clutch size, reproductive success) and adult body condition. Also, to test whether mute swans selected their territories non-randomly (some territories less preferred and some more preferred than expected under random territory choice), we compared the distribution of swan territory occupancy to the Poisson distribution, which assumes a pattern of random territory selection (Sergio & Newton, 2003).

## Material and Methods

### Study area and species

**Figure 1:**
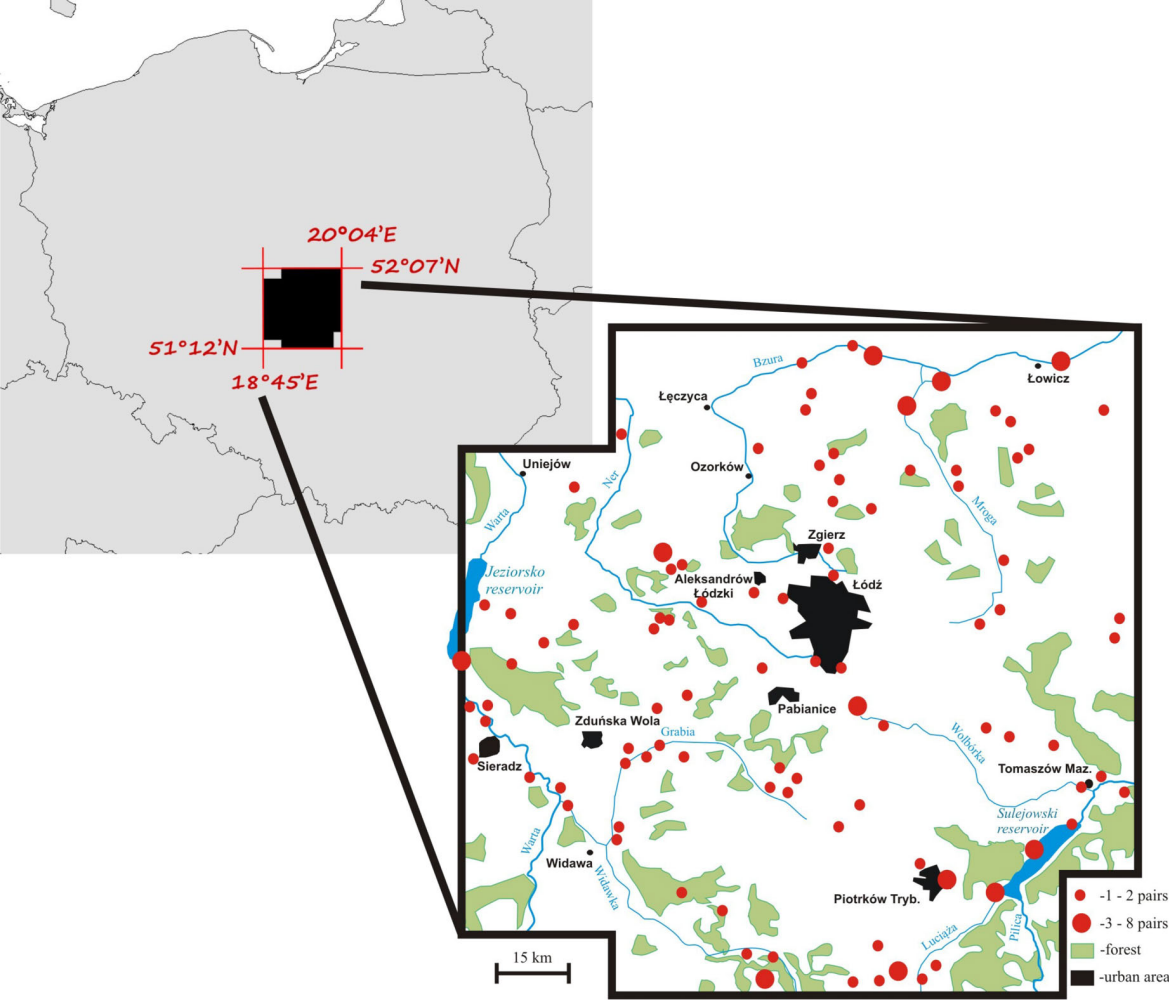
A map of the mute swan breeding territories within the study area, central Poland.

Territory occupancy by mute swans was assessed over the period of 19 years (1997–2015) within an area of 8,800 km^2^ in central Poland (Fig. 1). Swans from the local breeding population occupy habitats characterized by a varying degree of anthropogenic pressure. Most pairs nest at artificial reservoirs, mainly fishponds with intensive carp production, small ponds used for recreation, and dam reservoirs. Occupation of natural nesting-sites, such as peat bogs, oxbow lakes and flooded meadows is much less frequent (17% of all territories). All breeding birds leave their territories for winter (Włodarczyk et al., 2013), although their average migration distance is very short (81 ± 9.5 [SE] km, n = 186). The size of the study population was 70–100 pairs (Fig. 1) and it did not show considerable fluctuations among years (Włodarczyk & Janiszewski, 2007), which is consistent with the general stable population trend in Poland (Fig. S1). The majority of pairs nested solitarily on isolated waterbodies (79.3%). Up to 12 pairs nested at large dam reservoirs (max. 42.3 km^2^ of water surface), but even in such situations each pair occupied a distinct territory defended from conspecifics. Semi-colonial nesting reported for other populations (Wieloch, Włodarczyk & Czapulak, 2004) has not been recorded.

### Territory occupancy

Occupancy was assessed for 271 breeding swan territories. Due to the relatively low abundance of potential nesting sites (waterbodies) in the study area, we were confident to detect over 90% of all territories in our study area. Each territory was classified as occupied in a given year if a nest was recorded. Only territories that were continuously visited during at least 10 years (max. 19 years) were used in the analysis (n = 222). Occupation rate was calculated as the proportion of years when swans occupied the territory to the number of years when the territory was available for breeding. Some territories were unavailable for swans for a part of the study period, mainly due to human activity (e.g. drained or devoid of reed vegetation). For the purpose of some analyses (comparison to the random Poisson distribution) and presentation, territory occupancy was grouped into five categories: (1) very low occupancy (1–20% of years), (2) low occupancy (21–40% of years), (3) medium occupancy (41–60% of years), (4) high occupancy (61–80% of years), and (5) very high occupancy (81–100% of years).

Observations of marked individuals (n = 123) suggested that there was a non-negligible turnover of birds within territories during the study period. The median length of territory occupancy by a marked individual was three years and individuals that nested within the same territory for longer than eight years were recorded sporadically (7.3% of all individuals; Fig. S2A). Although we could not accurately estimate turnover rate of birds in the territories, as many individuals were unmarked, our data indicates that most territories characterized by high occupation rate were independently chosen by multiple pairs/individuals throughout the study period. Territories characterized by an over 10-year occupancy were occupied by marked individuals for an average of 35.6 ± 0.035 [SE]% of years, suggesting that each territory changed an owner ca. two times during the study period (Fig. S2B).

### Reproductive performance

Information on the reproductive performance of swans was collected since 1998. Each year, field observations started in late March, when pairs established territories, and finished in September, when first pairs left their territories with fledglings. Each occupied territory was visited at least four times during each breeding season. During these visits we collected data on the timing of laying, clutch size, and reproductive success (number of young fledged). The timing of laying for each pair was determined either by visiting the nest during laying or by a water-test developed for the mute swan, where the incubation stage is established with ±5 day accuracy by the way the egg floats in water (Czapulak, 2002). In total, reproductive data were collected for 1,345 breeding attempts of swans.

### Adult condition

We also captured adult birds within breeding territories to collect information on their body condition. Catching, ringing and handling of birds was performed under the licence of the Polish Academy of Sciences, with an approval of the Ministry of the Environment in Poland (DLP-VIII-6713-21/29762/14/RN). In total, we captured 123 individuals (64 males and 59 females) occupying 75 different territories. All birds were sexed with cloacal examination as described by Baker (1993). We measured total forearm length with ±1 mm accuracy and weighed birds using balance scale with an accuracy of 100 g (0.8–1.0% of average body mass of the mute swan). We used body mass adjusted for structural size (mass-size residuals) as the condition index of breeding birds. To obtain this estimate, we extracted residuals from a full-factorial model of sex and forearm length regressed against body mass (*R*^2^ = 0.67, *F*_3,119_ = 83.30, *p* < 0.001).

### Statistical analyses

To test whether occupancy of territories was random we compared distribution of occupation rate with the Poisson distribution using *χ*^2^ test. Occupancy pattern would be similar to the Poisson distribution, if the territories were occupied randomly and independently of their previous history (Krebs, 1989). Relationship of territory occupancy with egg laying date and clutch size were analysed with the General Linear Mixed Models (GLMMs). As reproductive success was highly zero-inflated, we used the generalized linear mixed model with binomial distribution and logit link function to analyse the probability of successful reproduction (at least one fledgling raised vs. no fledglings) and we used GLMM to analyse reproductive success for successful pairs only. The identity of territory was entered as a random effect in each model to avoid pseudoreplication resulting from repeated measures of the same pairs breeding repeatedly in the same place. The effect of year was also entered as a random factor and laying date was entered as a covariate in the analyses of clutch size and reproductive success. GLMM with territory identity included as a random factor was also used to analyse relationship between occupation rate and size-adjusted body mass of swans. In this model we entered the date of capture as a covariate, but due to the low sample size of captured birds in some years we did not control for between-seasonal variation. All GLMMs were analysed with JMP 12.1 (SAS Institute Inc., Cary, NC, USA). All values are presented as means ± SE.

## Results

**Figure 2:**
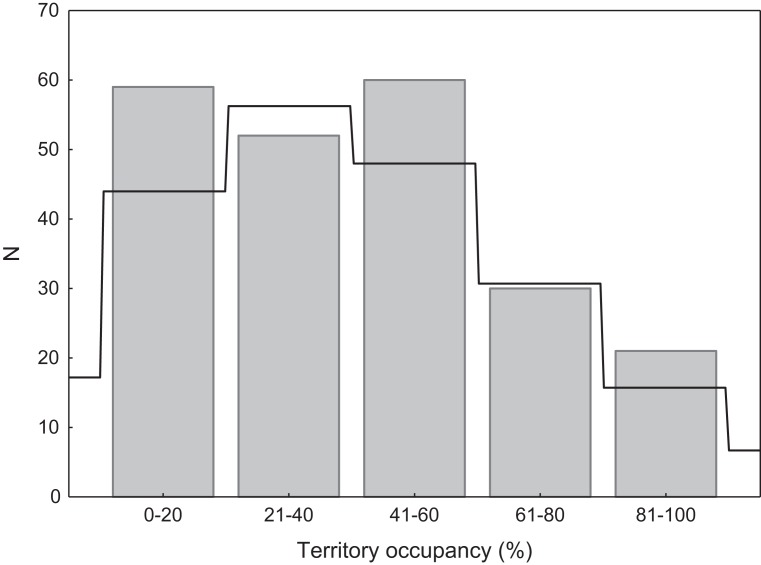
Distribution of territory occupancy by the mute swan. Expected random occupancy (according to the Poisson distribution) is marked with a black line.

We found that territories were randomly occupied by swans, as the distribution of occupation rate did not differ significantly from the expected Poisson distribution (*χ*^2^ = 4.36, *p* = 0.22; Fig. 2). Consistent with the Poisson distribution, territories with low or very low occupancy were significantly more frequent than territories with high or very high occupancy (49.8% vs. 22.9%; *G* = 17.76, *p* < 0.001; Fig. 2). There were no differences in occupancy between natural and artificial breeding sites (*t* = 1.26, *df* = 220, *p* = 0.20).

**Figure 3:**
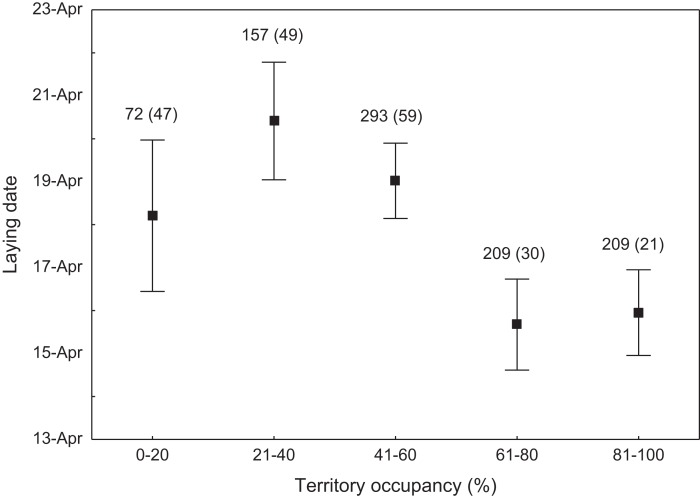
Laying date of mute swans nesting in territories of different occupancy. Number of reproductive events and number of territories (in parentheses) are shown for each occupancy category above the bars. Means ± 95% CI are presented.

**Figure 4:**
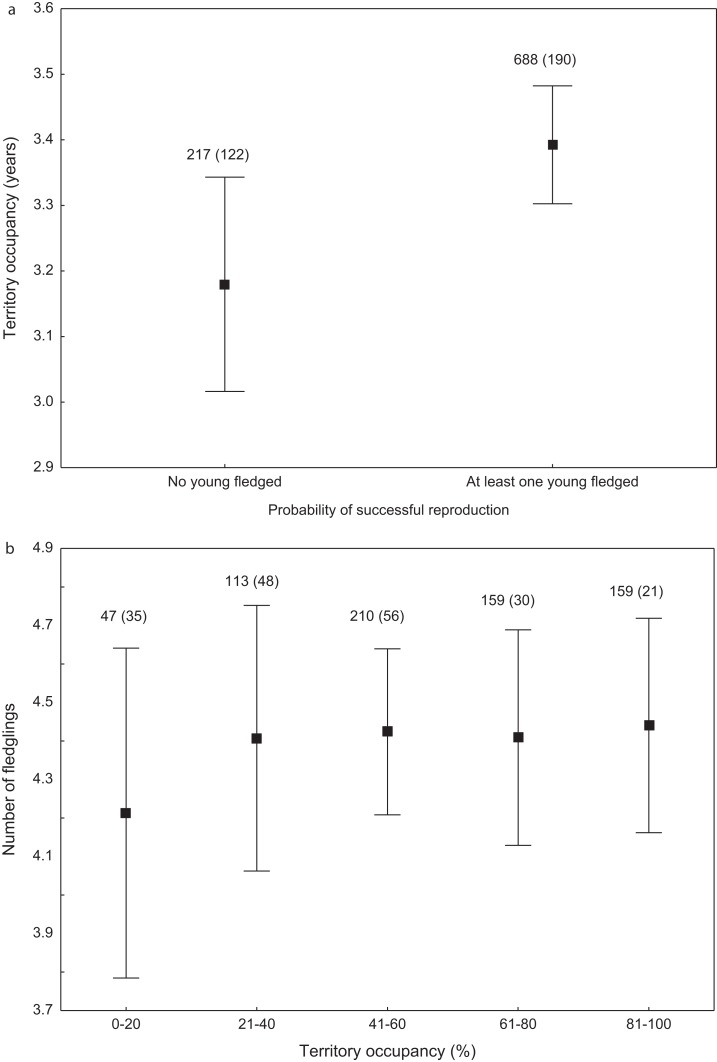
Probability of successful reproduction (A) and reproductive success of successful pairs (B) in mute swans nesting in territories of different occupancy. Number of reproductive events and number of territories (in parentheses) are shown for each occupancy category above the bars. Means ± 95% CI are presented.

**Figure 5:**
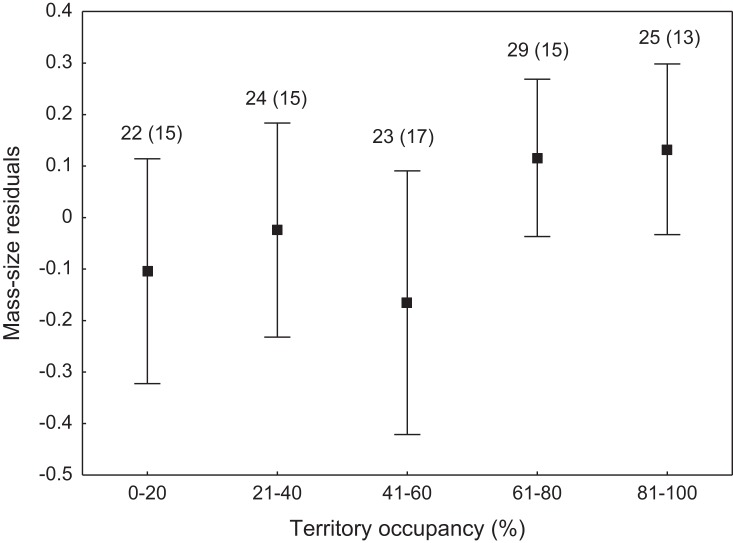
Body condition of adult mute swans (mass-size residuals) nesting in territories of different occupancy. Number of individuals and number of territories (in parentheses) are shown for each occupancy category above the bars. Means ± 95% CI are presented.

We found that the onset of egg laying was significantly related with territory occupancy, as birds bred earlier in the territories with higher occupancy (*F*_1,715_ = 6.96, *p* = 0.009, Fig. 3). On average, swans bred 3–5 days earlier in territories with high or very high (60–100%) occupancy when compared with territories characterized by low or very low (0–40%) occupancy (Fig. 3). While controlling for laying date, we did not find any significant relationship between occupation rate and clutch size (*F*_1,706_ = 2.79, *p* = 0.096). Although we found a significant relationship between territory occupancy and the probability of successful reproduction (*χ*^2^ = 4.64, *df* = 682, *p* = 0.031), this was due to the differences in laying date between territories of different occupancy. After accounting for the effect of laying date in the model the relationship lost significance (*χ*^2^ = 2.98, *df* = 681, *p* = 0.08; Fig. 4A). Territory occupancy was not related with reproductive success of successful pairs, either while controlling for the confounding effect of laying date (*F*_1,681_ = 0.02, *p* = 0.87; Fig. 4B) or while excluding laying date from the model (*F*_1,682_ = 0.55, *p* = 0.46). After controlling for the date of capture (*p* < 0.05), we did not find any relationship between territory occupancy and condition of adult birds measured with mass-size residuals (*F*_1,47_ = 0.54, *p* = 0.48; Fig. 5).

## Discussion

In this study we provided empirical evidence for a random and non-adaptive pattern of territory selection in the mute swan. We recorded relatively few territories which were occupied continuously throughout the study period, and there was a large proportion of territories that were occupied ephemerally, which was consistent with the assumption of random territory selection. Although swans settled slightly earlier in territories with high occupation rate, confirming their larger attractiveness, we found that reproductive success of swans did not vary with territory occupancy. Non-adaptive territory selection suggests that at the time of territory selection swans might not be able to reliably assess the fitness return associated with preferred territories.

In birds, territory occupancy has often been associated with direct and indirect measures of habitat quality and, thus, was suggested to reflect habitat selection (Arlt & Pärt, 2007). Most studies on territory occupancy have indicated non-random patterns of territory selection, where certain territories (usually high-quality ones) were preferred, while others were avoided (e.g. Sergio & Newton, 2003). Under this pattern, the frequency of territories with high occupation rate should be higher than expected from the random Poisson distribution, and such a situation has been reported for a variety of species, e.g. eagle owl *Bubo bubo* (Marchesi, Sergio & Pedrini, 2002), black kite *Milvus migrans* (Sergio & Newton, 2003), and white stork *Ciconia ciconia* (Janiszewski, Minias & Wojciechowski, 2013). By contrast, we found that territory occupancy in the mute swan conformed to the Poisson distribution, indicating a random pattern of territory selection. We observed a higher number of territories occupied ephemerally compared to territories occupied regularly. This implies that birds did not show strong preferences towards certain territories, which were likely to provide the highest fitness payoff.

The laying date was the only reproductive trait that was related to territory occupancy in our population of the mute swan and we found that swans bred earlier in the territories characterized by higher occupancy. In many migratory species of birds, there is a phenotype-dependent competition for early arrival at breeding grounds (Møller, 1994). High-quality individuals are usually able to prepare more quickly for spring migration, migrate at a higher speed, and consequently, arrive at breeding grounds earlier than poor-quality conspecifics (Aebischer et al., 1996; Hotker, 2002; Elmberg et al., 2005; Janiszewski, Minias & Wojciechowski, 2014b). As many territories are not yet occupied early in spring, first-arriving individuals are able to choose the most attractive sites and initiate breeding earlier, which may provide additional reproductive benefits. Thus, a positive correlation between laying date and territory occupancy is expected, as both these measures should be rather treated as proxies of preferences towards certain territories, rather than of adaptiveness of territory selection.

Despite the positive correlation between territory occupancy and the onset of breeding, we found no evidence for higher fitness return associated with settling in preferred territories. This mismatch between preferences and reproductive success strongly suggests that birds were not able to reliably assess territory quality and environmental cues used by swans for territory selection could poorly predict their reproductive payoff. We hypothesize that this non-adaptive territory choice by the mute swan could be possibly attributed to the exceptionally long reproductive cycle of this species. Entire reproductive cycle from egg laying to young fledging takes over four months in the mute swan (Wieloch, Włodarczyk & Czapulak, 2004) and environmental or ecological conditions within breeding territories are likely to change considerably during this period. Mute swans establish territories early in spring, usually in March, and it might be very difficult for swan pairs to reliably assess profitability of territories at the time of settlement. A similar explanation for the mismatch between territory preferences and fitness has been proposed for the water pipit *Anthus spinoletta*, in which early arrival at alpine breeding grounds impeded an assessment of territory quality (Bollmann, Reyer & Brodmann, 1997). Although early arriving individuals gained easy access to vacant territory, the presence of snow cover at the time of territory establishment did not allow reliable prediction of annual reproductive success on the basis on environmental cues (Bollmann, Reyer & Brodmann, 1997). Non-adaptive territory selection has also been reported for several other passerines. In the Brewer’s sparrow *Spizella breweri*, only offspring body condition positively correlated with the preferred habitat type, while other reproductive traits were randomly distributed among the habitats of varying attractiveness (Chalfoun & Martin, 2007). Poor temporal predictability of habitat characteristics also explained the mismatch between habitat preferences and both reproductive performance and survival in the northern wheatear *Oenanthe oenanthe* (Arlt & Pärt, 2007). Although all these passerine species have short reproductive cycles (ca. one month), the uncoupling of preference and habitat quality has been attributed to rapid habitat alterations. For example, short field layer at the time of young rearing was critical for the northern wheatear to achieve high reproductive success, but ca. 50% of preferred sites with a short field layer at the time of selection grew a tall field layer at the time when nestlings were being fed (Arlt & Pärt, 2007). Although habitat alterations within mute swan territories might not be so rapid, it is likely that exceptionally long parental care may considerably reduce temporal correlation between the conditions at the time of territory establishment and conditions during the critical stages of brood care.

Non-adaptive territory choice by the mute swan was also supported by the lack of relationship between territory occupancy and body condition of breeding adults. Nutritional condition of breeding pairs or their offspring primarily depends on the availability of food supply within territories (Rotenberry & Wiens, 1998; Solonen, 2011). High quality territories should guarantee rich food resources that allow territory owners to maintain their body in good condition throughout the entire breeding season. Our results suggest that environmental cues used for territory selection by mute swans could poorly predict not only brood safety, but also feeding conditions on the later stages of reproduction. Mute swans preferably feed on submerged vegetation, which starts to develop after their arrival at the breeding grounds and availability of this food source during late spring and early summer months might be difficult to predict at the time of territory establishment (Wieloch, Włodarczyk & Czapulak, 2004). Owing to their large body size and considerable energetic demands, swans can also cause severe depletion of water plants due to overgrazing (Wood et al., 2012). This can be a serious problem, especially at the sites where flocks of non-breeding swans gather for moult before reproductive activities of breeding pairs are concluded.

Finally, low predictability of territory quality in the mute swan can be caused by human activities. Mute swans across the entire breeding range often nest in the anthropogenic landscape (Wieloch, Włodarczyk & Czapulak, 2004) and in our population only 17% of pairs nested in natural habitats. In general, anthropogenic habitats are likely to show high level of unpredictability due to human-related disturbance that is not linked to environmental factors. In consequence, there is often a mismatch between preferences or cues used by individuals during territory selection and the true quality of anthropogenic habitat (Kokko & Sutherland, 2001). For example, lapwings *Vanellus vanellus* often choose lush green fields for breeding but modern farming practise is based on common use of fertilizers that causes intensive plant growth. As a result birds abandon nests located at fields where cereals grow rapidly (Galbraith, 1989). Modern forest management has created open areas highly preferred by red-backed shrikes *Lanius collurio*, despite the fact that pairs which settle in this novel environment suffer much higher brood losses than pairs nesting in traditionally occupied farmland areas (Hollander et al., 2011). In sage sparrows *Amphispiza belli*, an inverse relationship between habitat preferences and reproductive success has been produced by redistribution of predators (mainly snakes) due to anthropogenic landscape-level changes (Misenhelter & Rotenberry, 2000). We suggest that unpredictable human-related activities may strongly determine reproductive output in our mute swan population. Water level in fish ponds is adjusted to the needs of carp production and we have recorded cases where ponds were intentionally drained during the breeding season, causing extensive losses of swan broods. Some attractive territories characterized by high occupation rate were repeatedly drained in successive breeding seasons, resulting in zero reproductive success of swan pairs, which were unable to predict these rapid habitat alterations. Other pairs were subject to strong disturbance associated with human recreation, but these activities intensified in early summer and were virtually unpredictable for birds during territory establishment. We have also recorded acts of vandalism, where adults or their offspring were deliberately killed, or the nests were destroyed by humans. Other unpredictable human-related causes of adult mortality and consequent brood losses included collisions with electricity wires and cars, predation by dogs, fish-line entanglement or fish-hook swallowing, and lead poisoning.

In conclusion, our study demonstrated a non-adaptive territory selection in a central-Polish mute swan population, where territory preferences measured with a long-term territory occupancy mismatched reproductive success and body condition of birds. We suggest that inability of swans to reliably assess territory quality was primarily caused by: 1) exceptionally long period of parental care, which reduces temporal correlation between the conditions at the time of territory selection and conditions during the critical stages of brood care; and 2) unpredictability of human-related activities that had a major impact on reproductive output of swan pairs in our population.

## Supplemental Information

10.7717/peerj.1852/supp-1 Supplemental Information 1 Long-term population trend for the Mute Swan in Poland. Data from the Monitoring of Birds of Poland (MBP) commission by the General Inspectorate of Environment Conservation in Poland.

10.7717/peerj.1852/supp-2 Supplemental Information 2 Length of territory occupancy by individual (marked) mute swans (a) and the relative territory occupancy (number of years occupied by a marked individual/total number of years occupied) in the population of the mute swan in central Poland.

10.7717/peerj.1852/supp-3 Supplemental Information 3 Raw data. Raw data.

## Additional Information and Declarations

### Data Deposition

The following information was supplied regarding data availability: The raw data has been supplied as a Supplemental Dataset.
