# Supplementary figures and images for: Non-adaptive territory selection by a bird with exceptionally long parental care
Source: PeerJ. 2016 Mar 24;4:e1852. doi: 10.7717/peerj.1852 (PMC4811171; doi:10.7717/peerj.1852)

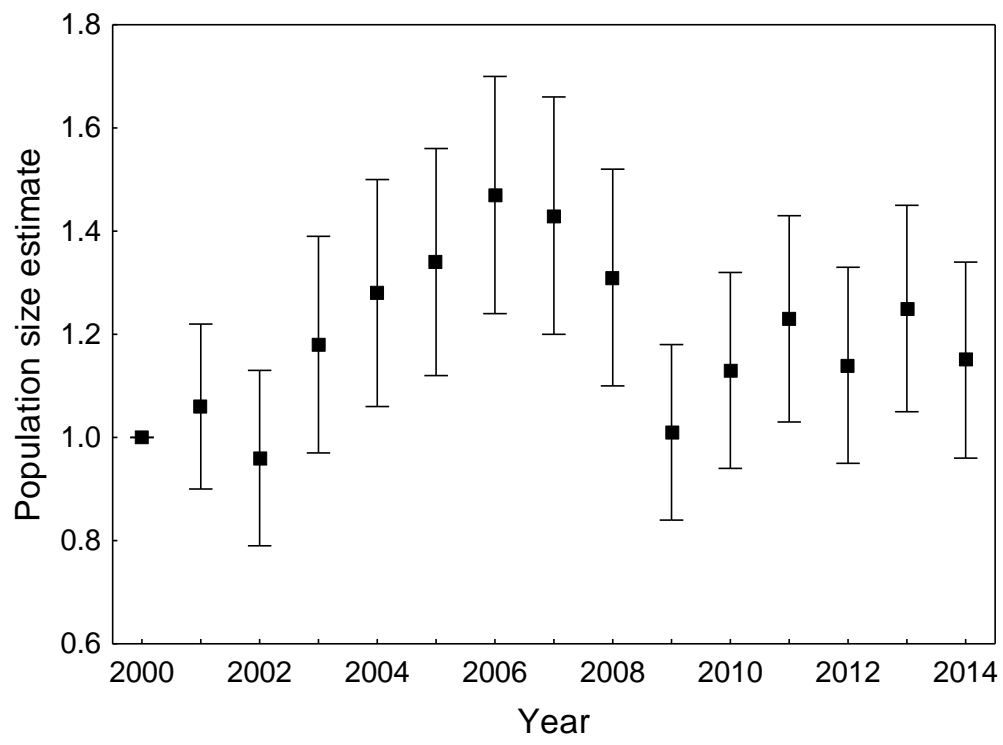

Supplement: Supplemental Information 1 [file peerj-04-1852-s001.pdf]

**a**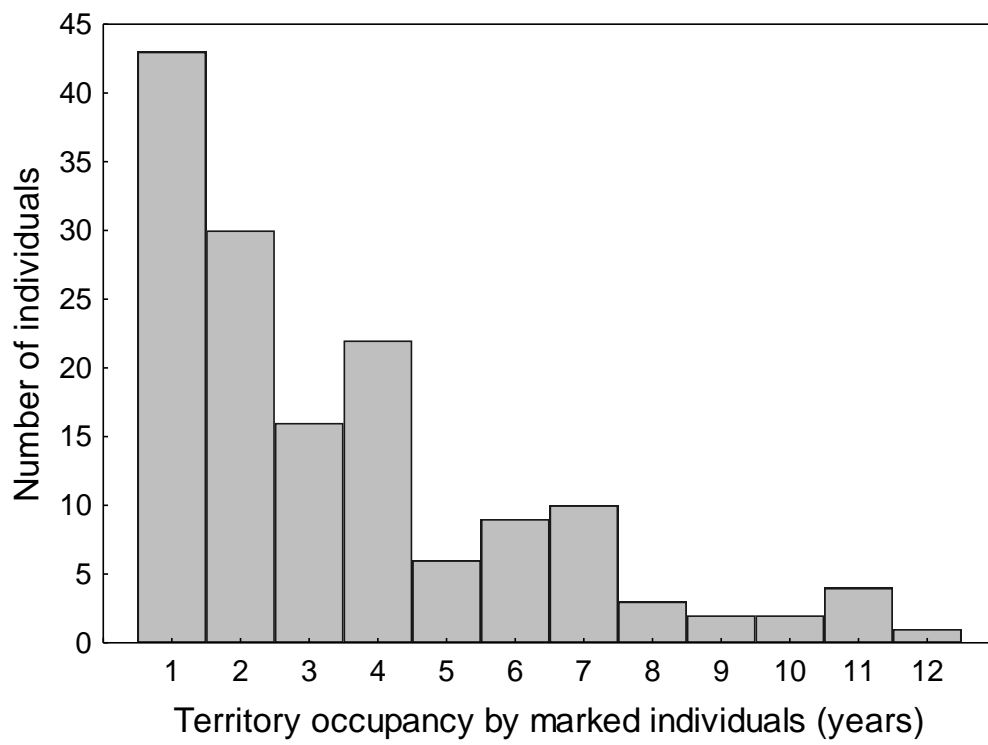**b**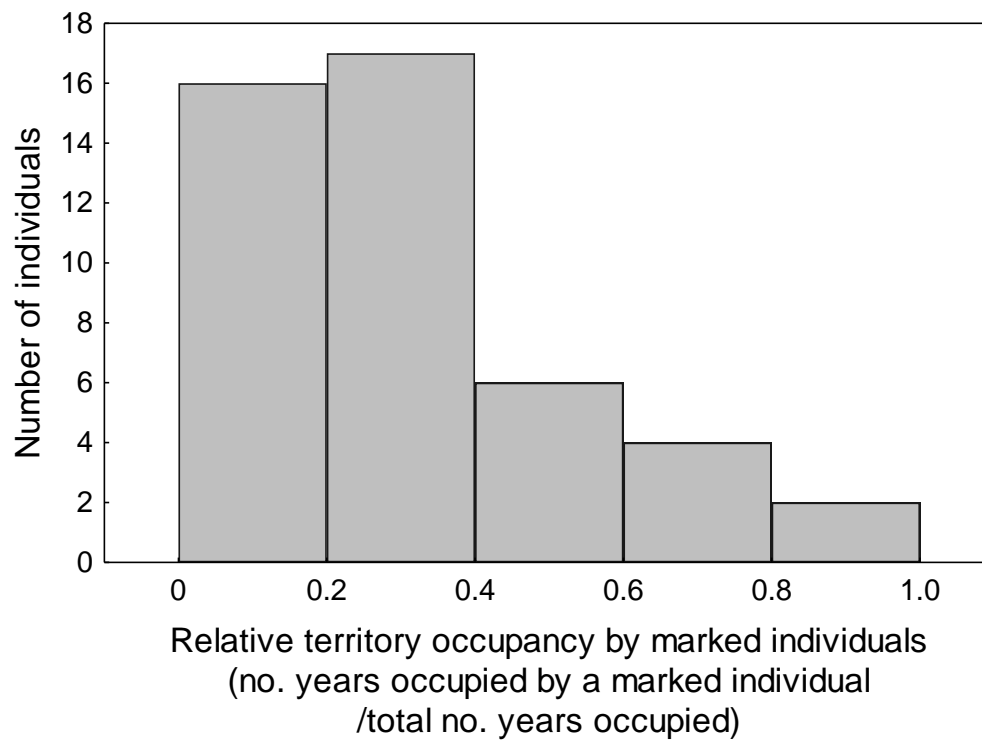

Supplement: Supplemental Information 2 [file peerj-04-1852-s002.pdf]
